# Optimization of clustering parameters for single-cell RNA analysis using intrinsic goodness metrics

**DOI:** 10.3389/fbinf.2025.1562410

**Published:** 2025-06-11

**Authors:** Nicolina Sciaraffa, Antonino Gagliano, Luigi Augugliaro, Claudia Coronnello

**Affiliations:** ^1^ Advanced Data Analysis Group, Ri.MED Foundation, Palermo, Italy; ^2^ Department of Economics, Business and Statistics, University of Palermo, Palermo, Italy; ^3^ National Center for Gene Therapy and Drugs based on RNA Technology, Palermo, Italy

**Keywords:** single-cell, clustering, intrinsic metrics, ElasticNet, robust linear mixed regression

## Abstract

**Introduction:**

The accurate clustering of cell subpopulations is a crucial aspect of single-cell RNA sequencing. The ability to correctly subdivide cell subpopulations hinges on the efficacy of unsupervised clustering. Despite the advancements and numerous adaptations of clustering algorithms, the correct clustering of cells remains a challenging endeavor that is dependent on the data in question and on the parameters selected for the clustering process. In this context, the present study aimed to predict the accuracy of clustering methods when varying different parameters by exploiting the intrinsic goodness metrics.

**Methods:**

This study utilized three datasets, each originating from a distinct anatomical district and with a ground truth cell annotation. Moreover, the investigation employed two clustering methods: the Leiden and the Deep Embedding for Single-cell Clustering (DESC) algorithm. Firstly, a robust linear mixed regression model has been implemented in order to analyze the impact of clustering parameters on the accuracy. Consequently, fifteen intrinsic measures have been calculated and used to train an ElasticNet regression model in both intra- and cross-dataset approaches to evaluate the possibility of predicting the clustering accuracy.

**Results and discussion:**

The first-order interactions demonstrated that the use of the UMAP method for the generation of the neighborhood graph and an increase in resolution has a beneficial impact on accuracy. The impact of the resolution parameter is accentuated by the reduced number of nearest neighbors, resulting in sparser and more locally sensitive graphs, which better preserve fine-grained cellular relationships. Furthermore, it is advisable to test different numbers of principal components, given that this parameter is highly affected by data complexity. This procedure has enabled the effective prediction of clustering accuracy through the utilization of intrinsic metrics. The findings demonstrated that the within-cluster dispersion and the Banfield-Raftery index could be effectively used as proxies for accuracy, for an immediate comparison of different clustering parameter configurations.

## 1 Introduction

Single-cell RNA sequencing (scRNA-seq) captures the gene expression profiles of individual cells, enabling the identification and characterization of different cell populations within a sample through transcriptomics (Jovic et al., 2022). Accurate cell subpopulations identification is essential for many downstream analyses, and the ability to break down cell subpopulations relies on unsupervised clustering. The most widely used clustering methods for analyzing single-cell data are Louvain and Leiden algorithms. These methodologies depict cellular structures as neighborhood graphs, wherein densely connected modules are designated as clusters. However, the accuracy of such approaches may be constrained by the quality of the underlying graph, potentially leading to the inadvertent identification of spurious structures that do not align with the intrinsic characteristics of the data (Quah and Hemberg, 2022). Other algorithms that are frequently employed are those based on k-means. The K-means algorithm is inherently susceptible to local minima due to the sensitivity of the centroid estimation process to the initialization. The SC3 package addresses this issue by integrating additional results after running K-means repeatedly (Kiselev et al., 2017). However, its current implementation is optimized for smaller data sets and cannot be easily used with other methods outside the SC3 package (Patterson-Cross et al., 2021).

Deep learning has led to the introduction of numerous methodologies for the purpose of clustering scRNA-seq data. According to the nature of the neural network they are based on, these methods can be grouped in self-optimization-based methods, generative adversarial network-based methods, subspace clustering-based, Gaussian mixture model-based methods, spectral clustering-based methods (Liu et al., 2024). A recent comparison of these methods has revealed that the Deep Embedding for Single-cell Clustering method (DESC) has demonstrated superior performance in terms of clustering specific celltypes and capturing celltype heterogeneity (Liang et al., 2024).

A number of disadvantages were observed with both classical clustering and deep learning based methods. The inefficiency of classical clustering methods is attributable to the sparsity and high dimensionality of scRNA-seq data (Vasighizaker et al., 2022). Furthermore, deep learning methods demonstrated sub-optimal efficiency when accurately characterising cell relationships in heterogeneous populations, due to distortion in gene expression induced by biological variability and technical errors (Yao et al., 2024). To address these challenges, manifold based approaches have been recently proposed (Vasighizaker et al., 2022). Methods like scAMF (Single-cell Analysis via Manifold Fitting) fits a low-dimensional manifold within the ambient space and unfolds the data accordingly, compared to the traditional methods based on low-dimensional data representation (Yao et al., 2024).

Despite the numerous advances and adaptations of clustering algorithms, the optimal choice of the number of clusters remains a challenging and data-dependent process (Duò et al., 2018). Furthermore, all these algorithms require the input of parameters by the user, whose selection has a considerable impact on the resulting outcomes. For instance, the number of neighbors and the resolution influence the construction of the proximity graph and the scale at which clusters of cells are defined during the actual clustering process, respectively. In addition, the choice of dimensionality reduction approach affects the outcome of the clustering process by altering the distance between cells and reducing information.

Alongside the methodological considerations, the nature of the data itself requires examination. It is frequently the case that the number of cells present in a given sample is not fully known. Consequently, in the absence of knowledge regarding which specific types of cells are present, it is only possible to estimate the quality of the clusters obtained based on hypothesis and prior knowledge, with a tendency to underestimate the presence of unconventional, new, or rare cells (Jindal et al., 2018). A substantial proportion of publicly available droplet-based datasets contain cell type labels, which are typically inferred by clustering cells using scRNA-seq data. Consequently, any evaluation based on these labels is likely to be biased in favor of methods that are similar to the one used to derive the labels in the first place (Duò et al., 2018). To ensure the reliability of the cell labels, it is essential to utilize as ground truth those derived through biologically reliable methods e.g., FACS sorting, which have been manually curated and are independent of the annotation algorithms utilized for scRNA-seq analysis.

In the absence of prior knowledge regarding the specific cell type, it is possible to evaluate the quality of the cluster by employing intrinsic metrics alone. In contrast to extrinsic metrics, which utilize information pertaining to the known true split, intrinsic metrics do not make use of any external information and assess the goodness of clusters based solely on the initial data and the quality of the split (Han et al., 2012). These metrics have been used to evaluate various clustering methodologies, including the Silhouette index for scLCA (Cheng et al., 2019), the Calinski-Harabasz index for CIRD (Lin et al., 2017), and the Gap statistic for RaceID (Grün et al., 2015). However, these tools have been developed with the specific aim of identifying cells belonging to specific cell types. For instance, RaceID was developed to identify rare enteroendocrine cells in murine intestinal samples (Jindal et al., 2018). While these tools can be applied to any sample type, the context in which they were developed may influence their capacity to handle the complexities of different samples.

In this context, the present study sought to predict the accuracy of the clustering method when varying different parameters. In order to accomplish this objective, the following steps were taken. Firstly, the impact of various clustering parameters on the accuracy of cell clustering was assessed. Secondly, the accuracy of cell clustering was predicted through the intrinsic metrics. To this end, three reference datasets have been selected from those available for download as models in the CellTypist organ atlas (Domínguez Conde et al., 2022; Xu et al., 2023).

The significance of this work lies in its capacity to facilitate a more nuanced comprehension of the utilization of clustering parameters for the identification of discrete cell populations within the context of single-cell RNA-seq data, particularly in situations where no supervisory input is available. In the absence of information regarding the cell types under investigation, this analysis can assist in obtaining a cluster’s structure in a manner that is as truthful as possible. This prevents the lack of data from leading to the misuse of clustering parameters. The increased appropriateness of the analysis may facilitate the detection of complex and sometimes unknown cellular structures, thus promoting the discovery of new biological insights.

## 2 Materials and methods

**FIGURE 1:**
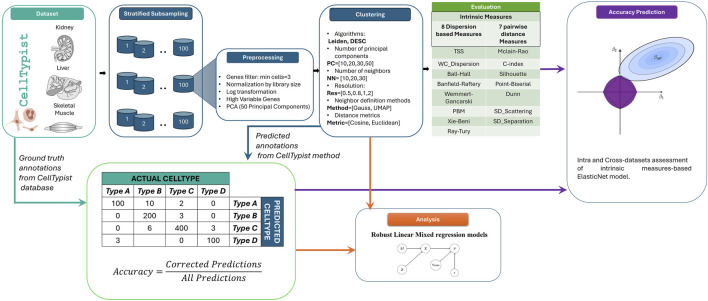
Workflow of clustering parameter analysis. Datasets were collected from the CellTypist database with their manually curated annotations (green). These data were subsampled, preprocessed and clustered using different methods and parameters (blue). The CellTypist method was used to predict the labels of the clustered dataset, and these predicted annotations were compared with the ground truth annotations from the CellTypist database to obtain the accuracy. These values were used to analyse how different parameters affect the accuracy (orange) and to predict the accuracy of the clustering method when different parameters are varied (purple).

In order to achieve the objective of this work, a simulation study was performed, as illustrated in Figure 1.

### 2.1 Datasets

The ground-truth annotations were downloaded from the CellTypist organ atlas (https://www.celltypist.org/organs). This ensures the utilization of a meticulously curated annotation for each viable cell, i.e., cells that have undergone death or damage have been filtered *a priori*. The employment of well-annotated viable cells also permits the circumvention of any bias that may be introduced by quality control procedures. In particular, the following datasets have been selected because they encompass diverse biological districts, thus representing different cell subpopulations, and because the non-normalized count matrices have been made available for subsequent analysis.

1. Liver organ from MacParland model (GSE115469). MacParland et al. identified 20 hepatic cell populations, from the transcriptional profiling of 8,444 cells obtained from liver grafts of five healthy donors, by flow cytometry, immunohistochemical examinations of human liver, analysis of differentially expressed genes, and examination of marker genes (MacParland et al., 2018). The 20 clusters have been identified with eight cell populations: six distinct hepatocyte populations, three endothelial cell populations, one as cholangiocytes, one as hepatic stellate cells, two as macrophages, three as T-cells, one as NK cell cluster, two as B-cells, and one as erythroid cells. The cell-type identities for each cluster were determined manually using a compiled panel of available known hepatocyte/immune cell transcripts.
2. Skeletal muscle from De Micheli Model (GSE143704). The authors collected the scRNA-Seq from surgically discarded tissue from 10 healthy donors undergoing reconstructive procedures and originating from different anatomical sites (De Micheli et al., 2020). A total of 22.058 cells were manually annotated and classified into 16 clusters comprising immune, vascular, stromal origin, myosatellite, and myofiber cells.
3. Kidney Cortex obtained from normal region of tumor-nephrectomy samples (https://explore.data.humancellatlas.org/projects/29ed827b-c539-4f4c-bb6b-ce8f9173dfb7). This data has been collected from the cortex of the kidneys of 18 donors. It consisted of 48.772 cells distributed among 47 clusters detailed in the reference.

### 2.2 Preprocessing

From each dataset, 20% of cells respecting the proportion of the original dataset (i.e., stratified subsampling) have been subsampled 100 times. For each subsampling, a standard single-cell analysis has been performed in Python environment through the Scanpy toolkit (Wolf et al., 2018). The preprocessing of scRNA-seq data usually starts with the assessment of its quality, removing low-quality cells (Luecken and Theis, 2019). However, since for the selected datasets good cells have already been filtered, this step has been skipped. Genes have been filtered when present in at least three cells according to common practice in the field. Data have been normalized by library size and log transformed, to rectify technical inconsistencies within the data set, such as disparities in sequencing depth between cells. Highly variable genes likely to be informative for further analysis have been selected and dimensionality reduction has been performed through principal component analysis. The scRNA-seq data are high-dimensional, with expression measured for thousands of genes. Dimensionality reduction is a pivotal step that transforms this high-dimensional data into a low-dimensional representation, facilitating the visualization and downstream analysis of the data.

### 2.3 Clustering algorithms

Two distinct algorithms have been used for the purpose of clustering single-cell data: the Leiden algorithm and Deep Embedding for Single-cell Clustering (DESC) (Li et al., 2020). The Leiden algorithm has been chosen since methods based on community detection have been shown to outperform scRNA-seq data clustering algorithms and it is the core strategy of widely used methods like Monocle3 and Seurat (Yu et al., 2022). It allows the detection of communities in the k-nearest-neighbor graph whose nodes reflect the cells in the dataset. In a low-dimensional space obtained through principal component analysis, the distance between cells has been computed and then the k most similar are connected (Wolf et al., 2019). In contrast, the DESC algorithm employs a deep neural network to construct a non-linear map from the original scRNA-seq data to a low-dimensional feature space where each cell is iteratively relocated to its nearest cluster.

### 2.4 Simulation parameters

A simulation study has been implemented in order to assess the effects of varying clustering parameters on accuracy. For each sample obtained by performing a stratified subsampling, both clustering algorithms have been executed, varying the following parameters for a total of 192 combinations.

• Number of principal components: PC = [10, 20, 30, 50]. Size reduction through principal component analysis (PCA) aims to capture the underlying trends and structures in the data by projecting observations into a reduced space that preserves as much variance as possible. In this case, it aims to systematically evaluate how variation in the number of PCs affects the quality of clustering, aiming to identify a configuration that effectively balances the reduction of dimensionality with the preservation of essential biological structure.
• Number of neighbors: NN = [10, 20, 30]. This parameter plays a key role in identifying the local environment of each cell within the reduced multidimensional space, i.e., constructing the graph structure on which clusters of cells are then identified by community identification algorithms.
• Resolution: Res = [0.5, 0.8, 1, 2]. Resolution plays a crucial role in determining the granularity of the identified clusters.
• Neighbor definition methods: Method = [Gauss, UMAP]. Depending on the method considered, the way of defining the global and local structure of the cells on which the clustering algorithms will be implemented changes. In Scanpy, the nearest neighbors distance matrix and a neighborhood graph of observations are computed using the UMAP (McInnes et al., 2018) or the Gauss method (Haghverdi et al., 2015).
• Distance metrics: Metric = [Cosine, Euclidean]. The choice of distance metric in methods such as Gauss or UMAP, used to identify nearest neighbors, is crucial because it defines how the 'proximity’ between points in the dataset is calculated, i.e., the distance of cells in gene expression space reduced by PCA. Different metrics, such as Euclidean distance or cosine, interpret proximity in different ways, directly influencing which points are considered neighbors. This has an impact on cluster formation, as the structure of the resulting groups depends on the perceived similarity between the data.

### 2.5 Accuracy computation

The clusters have been annotated using the CellTypist method. Therefore the accuracy has been computed comparing the labels obtained from the CellTypist method with those coming from the CellTypist database, which represents the ground truth. On the one hand, this choice guarantees the use of a well-curated annotation, against which the pipelines developed with the Scanpy toolkit have been tested. On the other hand, the use of CellTypist for both training and testing avoids the introduction biases due to labelling method.

### 2.6 Robust linear mixed regression model

A linear model with random effects, to take into account the effect of the subsampling, was applied to previously described data including Method, Metric, NN, PC, Res as categorical variables. The accuracy has been used as a dependent variable. To account for outliers in accuracy distribution, a Robust Linear Mixed regression model was implemented, using the function rlmer in the R package robustlmm, allowing for weighting observations that deviate significantly from the expected value of the distribution (Koller, 2016). A graphical summary of the robust linear mixed model is depicted in Supplementary Figure S1. The first-order interactions model has been analyzed to correctly interpret the effect of parameters on accuracy by removing possible bias effects. Theoretical and methodological details are described in Supplementary Material.

### 2.7 Intrinsic goodness metrics

The intrinsic goodness metrics evaluate a clustering procedure by examining how well the clusters are separated and how compact the clusters are without knowing the true labels. A total of 15 intrinsic measures were calculated, based on dispersion measures and intra- and inter-cluster pairwise distances.

The metrics based on dispersion measures are calculated from the total scatter matrix measuring the dispersion around the barycenter of the data matrix. In particular, the following have been calculated:

• Total sum of square (TSS) (Edwards and Cavalli-Sforza, 1965): The trace of the total scatter matrix is the sum of all the squared Euclidean distances from the data barycenter.
• Within cluster dispersion (WC_Dispersion) (Marriot, 1971): It has been one of the most common validity indices in clustering applications. The trace of the within-group scatter matrix, that is the sum of the squared distances between the observations and the barycenter of the cluster.
• Ball-Hall (Ball and Hall, 1965): This is the average version of the previous measure, that is the mean, through all the clusters, of their mean dispersion.
• Banfield-Raftery (Banfield and Raftery, 1993): This index is the weighted sum of the logarithms of the traces of the within-group scatter matrix of each cluster. Compared to other previous dispersion measures performing well when all the clusters have the same dispersion, this index is based on the cluster variances, therefore it tends to be more appropriate when the clusters are hyperspherical but of different sizes.
• Pakhira–Bandyopadhyay–Maulik PBM index (PBM) (Malay et al., 2004): This index considers both the total scatter matrix and the within-cluster dispersion, and is calculated using the distances between the points and their barycenters and the distances between the barycenters themselves. This ratio is normalized by the number of clusters, which was introduced to compensate for the growth of the dispersion ratio with further data partitioning.
• Wemmert-Gancarski (Bandyopadhyay and Saha, 2008): This index is defined as the weighted mean, for all the clusters, of the mean in each cluster of the quotient between the distance of this point to the barycenter of the cluster it belongs to and the smallest distance of this point to the barycenters of all the other clusters. It is based on a cluster score that accounts for the number of objects that are closer to their cluster centroid than to the centroids of other clusters.
• Xie-Beni (Xie and Beni, 1991): It is defined as the quotient between the mean squared distances of all the points concerning the barycenter of the cluster they belong to and the minimum of the minimal squared distances between the points in the clusters.
• Ray-Tury (Ray and Turi, 1999): It is defined as a quotient between the mean of the squared distances of all the points concerning the barycenter of the cluster they belong to and the minimum of the squared distances between all the cluster centroids.

The computed metrics based on intra- and inter-cluster pairwise distances, i.e., the measure of cluster cohesion and separation respectively, have been the following:

• Mclain-Rao (McClain and Rao, 1975): It is the ratio of the average intra-cluster to the average inter-cluster distance.
• C-index (Hubert and Schultz, 1976): It is a normalized sum of the distances between all the pairs of objects that belong to the same cluster; the normalization scheme is based on the minimum and maximum distance sums in the dataset.
• Dunn (Dunn, 1974): In contrast with the other indexes based on cluster pairwise distance, the Dunn index has the smallest distance between two objects from different clusters in the numerator and the maximum intra-cluster distance, in the denominator.
• Silhouette (Peter, 1987): It is among the most used indexes for cluster evaluation since it is easily interpretable in the range [-1,1]. It is based on the normalized difference between the smallest average distance of the object to the objects belonging to any other cluster and its average distance to the other objects of the same cluster. The silhouette of a sample is a mean value of silhouette values from this sample. Therefore, the silhouette distance shows to which extent the distance between the objects of the same class differs from the mean distance between the objects from different clusters. Values close to −1 correspond to bad clustering results, while values closer to one correspond to well-defined clusters. Finally, the global silhouette index is the mean of the mean silhouettes through all the clusters.
• Point-Biserial (Milligan, 1981): this index represents the point-biserial correlation between the pairwise distance matrix and a binary matrix consisting of 0/1 entries that indicate whether or not two objects are in the same cluster.
• SD (SD_Scattering and SD_Separation) (Halkidi et al., 2001): The SD index is finally defined as the weighted sum of average scattering (S) and total separation between clusters (D). The weight is equal to the value of D obtained for the partition with the greatest number of clusters. In this case, there are no different numbers of clusters, therefore S and D are considered separately as two different indexes.

To analyze the interrelationships between the intrinsic goodness metrics, we computed the Spearman correlation and performed the hierarchical clustering analysis. The results of these calculations are illustrated by means of the clustermap function contained within the Seaborn package.

### 2.8 ElasticNet model for accuracy prediction

The goodness metrics have been used to predict the accuracy of identification of each cell type. Since some goodness metrics are intrinsically correlated to each other, encountering multicollinearity, ElasticNet has been chosen as a linear regression model. In fact, the ElasticNet model exploits both l1 and l2 norm directly applied to the regularization coefficients (Zou and Hastie, 2005). It is a linear combination of both Ridge and LASSO regression where the former exploits the l2-norm to shrink the parameters toward zero, while the latter relies on the l1-norm to introduce sparsity among them (Antonacci et al., 2024). This combination allows for learning a sparse model where few of the weights are non-zero like Lasso, while still maintaining the regularization properties of Ridge (Pedregosa et al., 2011). The dataset has been normalized and the ElasticNet model has been trained twice: in leave-one-out cross-validation (i.e., intra-dataset) and in cross-dataset validation to analyze the stability of the relevant metrics. The alpha parameter, that is the constant that multiplies the penalty terms, has been optimized through 5-fold cross-validation. The l1 ratio parameter has been set to 0.5 as default. The performance of the model has been assessed by computing the R2 score and the Root Mean Squared Error (RMSE).

The intrinsic metrics most capable of generalising the results of the model, will be compared with two extrinsic metrics to assess their consistency with measures aware of ground truth labels. The ARI (Adjusted Rand Index) computes similarity between predicted and ground-truth clusters (Hubert and Arabic, 1985). The Normalized mutual information (NMI) is a measure of the similarity between two partitions (Danon et al., 2005). It is calculated by determining the mutual information of the two partitions, and then normalising this by the respective entropies of the partitions. Both metrics range from 0 to 1, with higher values indicating better agreement.

## 3 Results

### 3.1 The impact of clustering parameters on clustering accuracy

**FIGURE 2:**
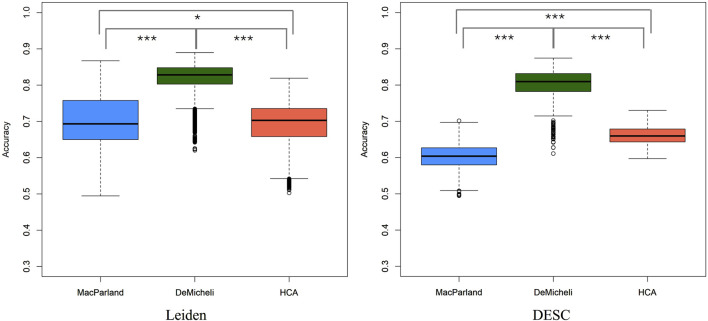
Boxplot of accuracy computed with all combinations of parameters for each dataset and both clustering algorithms. Statistics performed by Kruskal–Wallis test with Dunn’s post-hoc test,  *** p-value <2*10^−16^,  * p-value <10^−05^.

A robust linear mixed regression model was initially computed over five categorical variables (i.e. Method, Metric, Number of neighbors, Number of components, Resolution) for Leiden algorithm and on Number of neighbors and Resolution for DESC algorithm. The accuracy distribution for each dataset comprises 19.200 values, which are representative of the 100 subsampling for each of the 192 parameter combination remaining from Leiden algorithm, and 1.200 values for DESC, corresponding to 100 subsamples for each of the 12 parameter configurations. Figure 2 illustrates these distributions obtained through the two different clustering algorithms, demonstrating that there is a dataset effect, with the DeMicheli dataset exhibiting the highest accuracy compared to the other two datasets. Indeed, a comparison of the accuracy distributions of the three datasets using the non-parametric Kruskal–Wallis test revealed a significant difference between the distributions (p-value <2.2e-16). Subsequently, a Dunn’s post-hoc test with Bonferroni correction was conducted to identify specific differences between pairs of datasets. This revealed that significant differences were present in all compared pairs, as illustrated in Table 1.

**TABLE 1:** Results of Kruskal–Wallis test between the MacParland, DeMicheli, and HCA for both algorithms**.** Pairwise comparisons have been performed through Dunn’s post-hoc test (Bonferroni corrected).

Dataset Comparison	Kruskal–wallis leiden: χ^2^ = 31,700, p-value <2.2e-16 Kruskal–wallis DESC: χ^2^ = 2,898, p-value <2.2e-16			
	Algorithm	Z-Value	P.unadj	P.adj
DeMicheli - HCA	Leiden	156.50	2.2e-16	2.2e-16
	DESC	29.83	2.2e-16	2.2e-16
DeMicheli - MacParland	Leiden	151.77	2.2e-16	2.2e-16
	DESC	53.72	2.2e-16	2.2e-16
HCA - MacParland	Leiden	−4.73	2.212e-06	6.637e-06
	DESC	23.89	2.2e-16	2.2e-16

To determine the specific influence of each parameter on accuracy, while simultaneously accounting for potential biases or confounding effects resulting from interactions, a first-order interaction model was constructed for each dataset and the results are described hereby for each clustering method.

#### 3.1.1 Leiden algorithm

The residual plot and quantile-quantile (QQ) plot of the models are presented in Supplementary Figure S2. A visual inspection of the residuals indicated that they were normally distributed, with only minor anomalies present on the left tail. An exploratory analysis indicated that these values are primarily attributable to the utilization of the Gauss method, with Euclidean metric, resolution equal to 0.5 and number of neighbors equal to 30. This difference is more pronounced in DeMicheli dataset, where this configuration of parameters, combined with 50 principal components, causes anomalies in the left tail of the residuals. This observation justifies the use of a robust linear regression model. The normality test results, as presented in Table 2, corroborate the presence of these anomalies. However, the results also demonstrate that the first-order interaction models are an excellent fit for the datasets, with the linear predictors effectively explaining the observed variations in accuracy, as evidenced by the R^2^ values of 0.8891 for MacParland, 0.8190 for DeMicheli, and 0.9336 for HCA. The low bias and RMSE values provide further evidence of the model’s precision.

**TABLE 2:** Model evaluation metrics and results of normality tests. Model evaluation metrics (Bias, RMSE, and R^2^) and normality test results (Kolmogorov-Smirnov and Anderson-Darling) on the residuals of the first-order interaction model for the MacParland, DeMicheli, and HCA datasets for both clustering algorithms.

Alg	Dataset	Bias	RMSE	R^2^	Kolmogorov-smirnov statistic (D)	p-value	Anderson-darling statistic (A)	p-value
Leiden	MacParland	5.31e-05	0.0229	0.8891	0.011699	0.01044	3.5122	8.976e-09
	DeMicheli	0.00059	0.0164	0.8190	0.051781	<2.2e-16	117.82	<2.2e-16
	HCA	0.00012	0.0145	0.9336	0.011468	0.01282	5.2528	5.761e-13
DESC	MacParland	0.00054	0.0151	0.8320	0.04912	0.006112	4.5603	2.607e-11
	DeMicheli	0.00153	0.0186	0.8085	0.10584	4.212e-12	30.092	<2.2e-16
	HCA	0.00007	0.0108	0.7900	0.031748	0.1779	1.2395	0.003157

**FIGURE 3:**
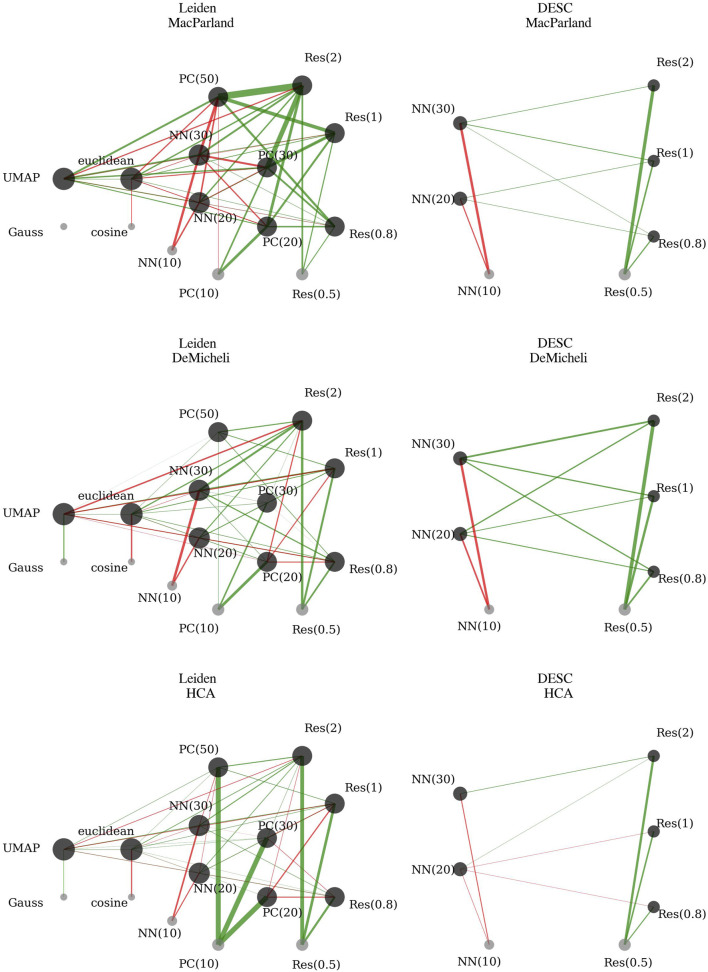
First-order robust linear mixed regression coefficients. Each network reports significant coefficients of robust linear mixed regression on accuracy for a given dataset. The nodes represent the different levels of categorical variables. The node dimension is proportional to the node degree, that is the sum of the significant interactions involving that node. In the absence of a link, it can be inferred that this parameter is not significant for that particular dataset. The green edges indicate that the parameter has a positive effect on accuracy in comparison to the reference values. Conversely, the red edges indicate that the parameter has a negative effect on accuracy in comparison to the reference values. The thickness of the edges is proportional to the magnitude of the effect. The reference values are: Method = Gauss; Resolution = 0.5; Number of Principal Components (PC) = 10; Number of Nearest Neighbors (NN) = 10; Metric = Cosine.

The outcomes of the first-order interaction model, as derived through robust linear mixed regression for each dataset, are detailed in Supplementary Tables S1–S3 and have been summarized in Figure 3, employing a network representation.

The Method variable coefficient (UMAP vs. Gauss) is significantly positive only for HCA and DeMicheli. Interactions involving UMAP indicate further significant effects:

• UMAP interacting with the Euclidean metric significantly increases accuracy across all datasets.
• Increasing resolution in combination with UMAP negatively impacts accuracy, contrasting with its positive effect when considered independently.
• UMAP interaction with principal components significantly enhances accuracy for MacParland and HCA datasets. In contrast, these interactions are weak or even detrimental in the DeMicheli dataset.
• UMAP interaction with nearest neighbors significantly boosts accuracy across all datasets.

Considering the magnitude of the coefficients, it is clear that using UMAP is preferable to Gauss across all three datasets. The overall effect of UMAP—driven by its strong positive interactions with metric, number of neighbors, and principal components—consistently outweighs the negative impact of its interaction with resolution, making it the most effective method overall.

Resolution coefficients are positively significant for all datasets, indicating improved accuracy when increasing clustering resolution from 0.5 to 0.8, 1, and 2. Interactions reveal more nuanced effects:

• Increasing resolution combined with the Euclidean metric further enhances accuracy, confirming a synergistic effect between these two parameters.
• Interactions between clustering resolution and the number of principal components significantly improve accuracy in the MacParland dataset. Similarly, for the DeMicheli dataset, the effect is consistently positive and increases with the number of components. In the HCA dataset, positive effects are only observed when combining higher resolution with a large number of components (e.g., 50), whereas interactions with 20 or 30 components show a negative or marginal contribution.
• Interactions between clustering resolution and the number of nearest neighbors consistently improve accuracy across all datasets, confirming the robustness of this relationship.

Considering the magnitude of the coefficients, the use of higher resolution levels—especially equal to 2—emerges as consistently beneficial across all datasets. The strong positive main effects, together with several substantial positive interactions, clearly outweigh the isolated negative effects found in interactions with UMAP. This confirms that increasing resolution enhances the ability to recover meaningful substructure in the data.

The number of principal components significantly increases accuracy for all datasets, with the strongest effect in HCA. However, for MacParland, the benefit declines beyond 30 components, and for DeMicheli, the improvement is minimal with 50 components. Interactions further illustrate complexity:

• Principal components interaction with the Euclidean metric increases accuracy for DeMicheli and HCA but decreases accuracy for MacParland.
• Principal components interaction with the number of nearest neighbors decreases accuracy in MacParland and HCA. In DeMicheli, the interaction effect is mostly neutral or slightly positive.

This complexity is also reflected in the magnitude of the coefficients. While the marginal effect of using 20 principal components is consistently strong—particularly in HCA—the overall benefit of increasing the number of components becomes clearer when accounting for interaction effects. Summing positive and negative contributions shows that a higher number of components enhances the clustering space and supports improved accuracy. However, while 50 components are optimal for both McParland and HCA (as shown by the highest cumulative positive coefficients), for DeMicheli the benefit plateaus at 30 components, with limited further improvement beyond that point.

The number of nearest neighbors negatively affects accuracy significantly across datasets. Further negative impact is observed when increasing the number of neighbors in combination with the Euclidean metric, suggesting that larger neighborhood sizes reduce clustering precision when Euclidean distances are used.

This interpretation is supported by the magnitude of the coefficients: since the baseline corresponds to 10 neighbors, the negative estimates for 20 and especially 30 neighbors indicate a consistent decline in performance in McParland and HCA. In DeMicheli, the difference is less pronounced—when summing marginal and interaction effects, the configuration with 30 neighbors appears slightly more favorable than 10. Nevertheless, the overall trend supports the use of smaller neighborhood sizes, as they tend to yield more accurate and localized neighborhood graphs, which in turn enhance the identification of cellular subpopulations.

The Euclidean metric coefficient is significantly negative across all datasets. Interactions indicate:

• UMAP combined with the Euclidean metric significantly enhances accuracy.
• Resolution increases paired with the Euclidean metric significantly boost accuracy.
• Euclidean metric combined with principal components has dataset-specific effects: positively for DeMicheli and HCA, negatively for MacParland.
• Increased nearest neighbors with the Euclidean metric further reduce accuracy across all datasets.

As with the number of neighbors, the marginal effect would suggest that the cosine metric is generally preferable. However, in DeMicheli and HCA, the negative baseline impact of the Euclidean metric is largely offset by its positive interactions with increased resolution and the use of UMAP, making it a reasonable choice in these contexts. The same cannot be said for MacParland, where—despite similar interaction patterns—the combination of parameters does not sufficiently counterbalance the negative marginal effect, ultimately favoring the cosine metric. Looking at the configuration of parameters to obtain the highest accuracy when all cells are used (Table 3), it can be seen that the cosine metric is preferable to Euclidean.

**TABLE 3:** Best and worst configurations for Leiden algorithm**.** For each dataset the configuration of parameters to obtain the highest value of accuracy using the whole number of cells has been also reported as *All cells* Type.

Dataset	Type	Metric	Method	PC	NN	Res	Predicted Accuracy *(mean ± std)*	Observed Accuracy
MacParland	Best	cosine	umap	50	10	2	0.823 ± 0.012	
	Worst	euclidean	gauss	50	30	0.5	0.503 ± 0.012	
	All cells	cosine	umap	30	10	2		0.839
DeMicheli	Best	euclidean	umap	30	30	2	0.864 ± 0.001	
	Worst	euclidean	gauss	10	30	0.5	0.698 ± 0.001	
	All cells	cosine	umap	30	10	2		0.882
HCA	Best	euclidean	umap	50	10	2	0.792 ± 4.4e^−4^	
	Worst	euclidean	gauss	10	30	0.5	0.540 ± 4.4e^−4^	
	All cells	cosine	umap	30	10	2		0.791

#### 3.1.2 DESC algorithm

Residual diagnostics reveal moderate deviations from normality and homoscedasticity, especially for MacParland and DeMicheli (Supplementary Figure S3). In both datasets, the Q-Q plots show pronounced left-tail deviations, and residuals versus fitted values indicate asymmetric spread, particularly for lower fitted values. These patterns are most prominent under the combination of 30 neighbors and resolution 0.5.

Normality test results confirm these observations. Kolmogorov-Smirnov and Anderson-Darling tests are significant for MacParland and DeMicheli, and partially for HCA, indicating deviations from normal residuals (Table 2). Despite this, model fit remains strong, with R^2^ values of 0.8320 for MacParland, 0.8085 for DeMicheli, and 0.7900 for HCA, thanks to the weighted fit. Bias and RMSE values are low across all models, supporting the overall reliability of the regression estimates.

Full model summaries are reported in Supplementary Tables S4–S6 and have been summarized in Figure 3, employing a network representation. Increasing resolution significantly improves accuracy across all datasets, with accuracy consistently rising as resolution moves from 0.5 to 2. The number of nearest neighbors negatively impacts accuracy across all datasets when considered alone, with the worst performance observed at 30 nearest neighbors. Interaction effects between resolution and nearest neighbors are generally positive for MacParland and DeMicheli datasets, highlighting enhanced performance when these parameters increase simultaneously. In the HCA dataset, however, interactions show mixed effects: negative or neutral at moderate resolutions (0.8 and 1) and positive only at the highest resolution level (2).

**FIGURE 4:**
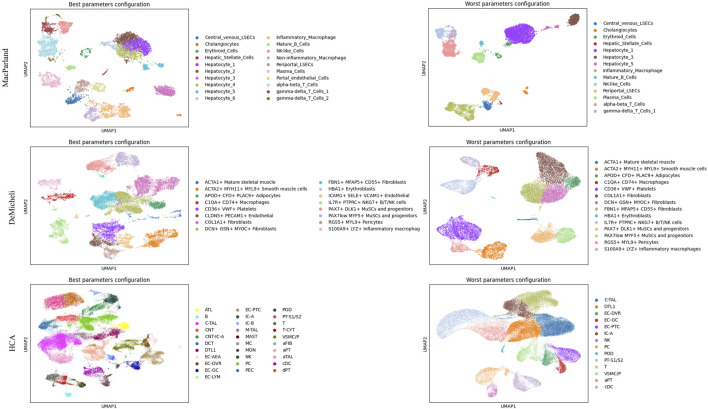
UMAP representation of Leiden results. For each dataset (in rows) the best (left column) and the worst (right column) configuration of parameters has been represented.

**FIGURE 5:**
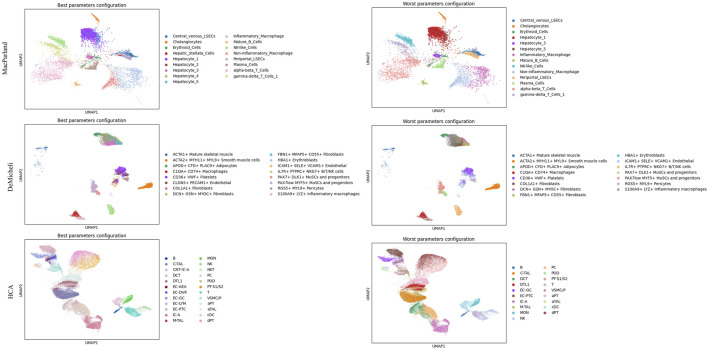
UMAP representation of DESC results. For each dataset (in rows) the best (left column) and the worst (right column) configuration of parameters has been represented.

This analysis enables the selection of the optimal and suboptimal parameter configurations for both Leiden and DESC algorithm, as illustrated in Table 3 and 4. The best and worst configuration (i.e., those configurations that allow for the prediction of the maximum and minimum mean accuracies, respectively) have been utilised to plot the whole datasets in UMAP space. The results of the Leiden algorithm are presented in Figure 4. It was observed that the optimal parameter configuration permitted the delineation of all 20 clusters for the MacParland dataset, all 16 clusters for the DeMicheli dataset, and 31 out of 47 clusters for the HCA dataset. Conversely, the most unfavourable configuration proved incapable of detecting all clusters. It is evident that a total of 6, 2 and 33 clusters have been missed for each dataset, respectively. The results of DESC algorithm (Figure 5) showed that the optimal parameter configuration permitted the delineation of 17 out 20 clusters for the MacParland dataset, all 16 clusters for the DeMicheli dataset, and 24 out of 47 clusters for the HCA dataset. Conversely, the worst configuration permitted the delineation of 14 out 20 clusters for the MacParland dataset, 15 out 16 clusters for the DeMicheli dataset, and 19 out of 47 clusters for the HCA dataset.

**TABLE 4:** Best and worst configurations for DESC algorithm.

Dataset	Type	NN	Res	Predicted Accuracy *(mean ± std)*
MacParland	Best	30	2	0.655 ± 0.02
	Worst	30	0.5	0.541 ± 0.02
DeMicheli	Best	10	2	0.849 ± 0.01
	Worst	30	0.5	0.725 ± 0.04
HCA	Best	10	2	0.691 ± 0.01
	Worst	30	0.5	0.628 ± 0.01

### 3.2 Prediction of clustering accuracy through intrinsic goodness metrics

**FIGURE 6:**
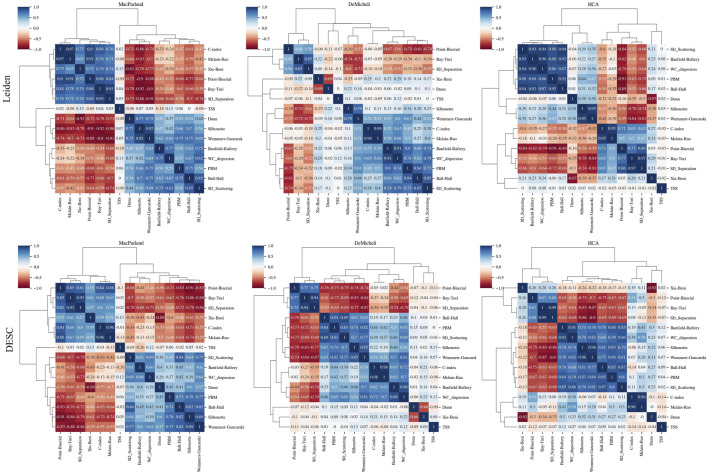
Feature correlation analysis. For each dataset (in columns) and for both clustering algorithms (in rows), each figure shows the correlation matrix heatmap calculated from the Spearman correlation and the dendrograms representing associations among the metrics. The length of the dendrogram branches represents the distance between metrics.

For each parameter combination, a total of fifteen intrinsic goodness metrics has been calculated. Figure 6 illustrates the interrelationships between the intrinsic goodness metrics through correlation and hierarchical clustering analysis. The heatmap of the correlation matrix demonstrates the existence of robust correlations between variables, exhibiting both positive and negative associations, which vary across datasets. The resulting dendrogram from the hierarchical clustering analysis reveals the existence of most of the clusters between variable are shared between the datasets and methods. In particular the following variables form a clear individual sub-cluster in all cases:

• C-index and Mclain-Rao are strongly (R > 0.95) positively correlated
• Silhouette index and the Wemmert-Gancarski are positively (R > 0.79) correlated
• Ball-Hall and PBM are positively (R > 0.71) correlated
• Banfield-Raftery and WC_Dispersion are positively (R > 0.66) correlated
• Point-Biserial, Ray-Turi and SD_Separation are positively (R > 0.49) correlated
• TSS is poorly correlated with other variables (R < 0.18).

In contrast, the remaining three intrinsic measures (SD_Scattering, Dunn, Xie-Beni) did not demonstrate a consistent pattern across the dataset and methods, and instead exhibited a tendency to join various sub-clusters. In every case Dunn and Xie-Bien are strongly anticorrelated (R > −0.66).

It is important to note that the observed association between these subclusters is contingent upon the specific dataset and methodological approach employed.

The 15 intrinsic metrics have been employed as features in the training of an ElasticNet regression model for the purpose of predicting accuracy values. The model has been trained using two approaches: an intra-dataset, i.e., leave-one-out cross-validation, and a cross-dataset approach. The resulting performances are presented in Table 5 in terms of R^2^ and RMSE values. With regard to the intra-dataset approach for Leiden algorithm, MacParland and HCA demonstrate the highest value in terms of R^2^, at 0.82 and 0.90, respectively. In comparison, DeMicheli exhibits a comparatively lower performance, with an R^2^ value of 0.64. In contrast, the RMSE values are comparable between the datasets with values lower than 0.0008. With regard to the cross-dataset approach, the results for MacParland and HCA are once again comparable, albeit with lower values for both R^2^ (0.72 and 0.71, respectively) and RMSE (0.1 and 0.14, respectively). DeMicheli results exhibit inferior performance (R^2^ = 0.54, RMSE = 0.18).

**TABLE 5:** Performance of the ElasticNet regression model for both clustering algorithms. Intra-Dataset performances have been reported in terms of mean ± standard deviation for R^2^ and Root Mean Squared Error (RMSE) for each dataset. Cross-dataset performances have a unique value for R^2^ and RMSE.

Algorithm		MacParland		DeMicheli		HCA	
		R^2^	RMSE	R^2^	RMSE	R^2^	RMSE
Leiden	Intra-Dataset	0.82 ± 0.08	0.0008 ± 0.0003	0.64 ± 0.07	0.0005 ± 0.0001	0.90 ± 0.03	0.0002 ± 0.0001
	Cross-Dataset	0.72	0.1	0.54	0.18	0.71	0.14
DESC	Intra-Dataset	0.76 ± 0.04	0.0002 ± 0.0002	0.73 ± 0.02	0.0002 ± 0.0002	0.64 ± 0.03	0.0003 ± 0.0002
	Cross-Dataset	0.61	0.13	0.53	0.23	0.57	0.13

The results obtained using the DESC algorithm are considerably lower than those obtained with Leiden algorithm. In relation to the intra-dataset approach, MacParland demonstrated the highest level of significance (R^2^ = 0.76). Conversely, DeMicheli exhibited comparatively diminished performance, with an R^2^ value of 0.73, while HCA exhibited the least efficient outcome, with an R^2^ value of 0.64. In contrast, the RMSE values are comparable between the datasets, with values lower than 0.0003. In relation to the cross-dataset approach, the outcomes of the model are low, with R2 equaling 0.61, 0.53, 0.57 and RMSE equaling 0.13, 0.23, 0.13, respectively, for the MacParland, DeMicheli and HCA dataset.

**FIGURE 7:**
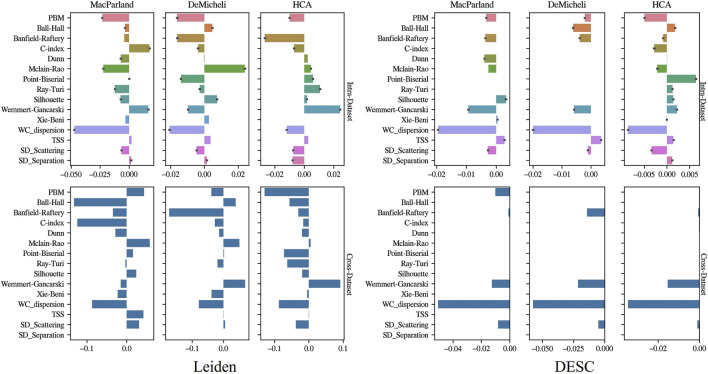
Feature selection results. Barplots show the value of ElasticNet coefficients in the case of the intra-dataset approach (first row) and cross-dataset approach (second row). Leiden results are depicted in the first column, DESC results in the second.

Figure 7 illustrates the ElasticNet coefficients estimated through both intra- and cross-dataset approaches. The bar charts demonstrate that, in the intra-dataset analysis, four out 15 intrinsic metrics were found to have ElasticNet coefficients different from zero and coherent among datasets and clustering algorithms: PBM, Banfield-Raftery, WC_dispersion, and SD_Scattering. The aforementioned features exhibited negative ElasticNet weights. The cross-dataset results corroborated the observation that Banfield-Raftery and WC_dispersion exhibited coherent behavior across datasets and algorithm.

**FIGURE 8:**
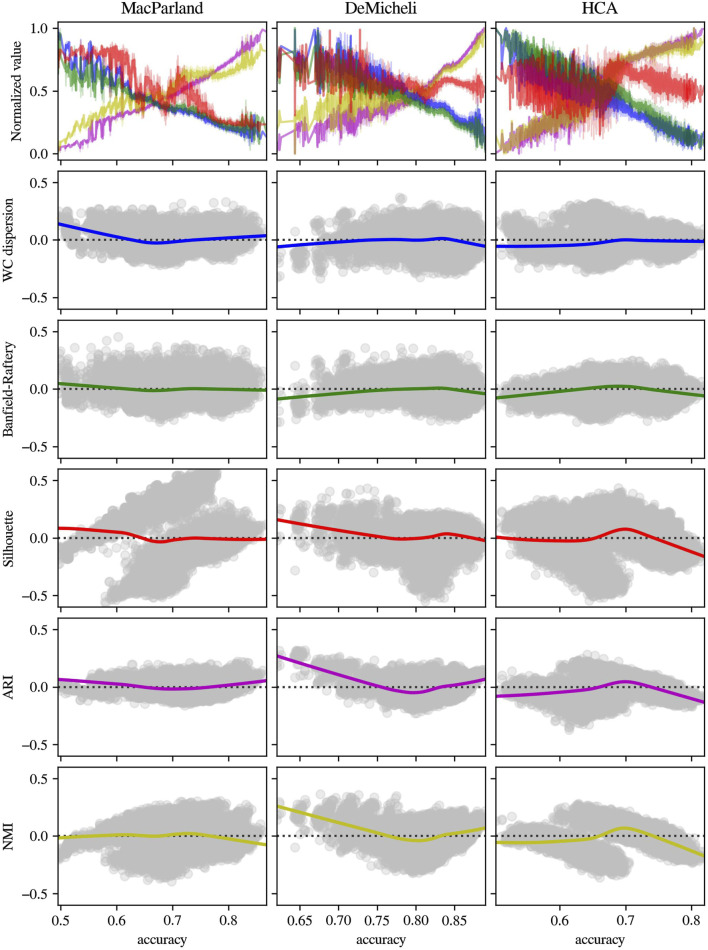
Distribution and residual plot of intrinsic and extrinsic metrics for Leiden algorithm outcomes. For each dataset (in columns), intrinsic measures (Banfield-Raftery in green, WC_dispersion in blue and Silhouette index in red) and extrinsic metrics (ARI in magenta and NMI in yellow) have been represented in terms of normalized distribution in function of accuracy (first line) and the scatterplot of the residual of linear regression.

**FIGURE 9:**
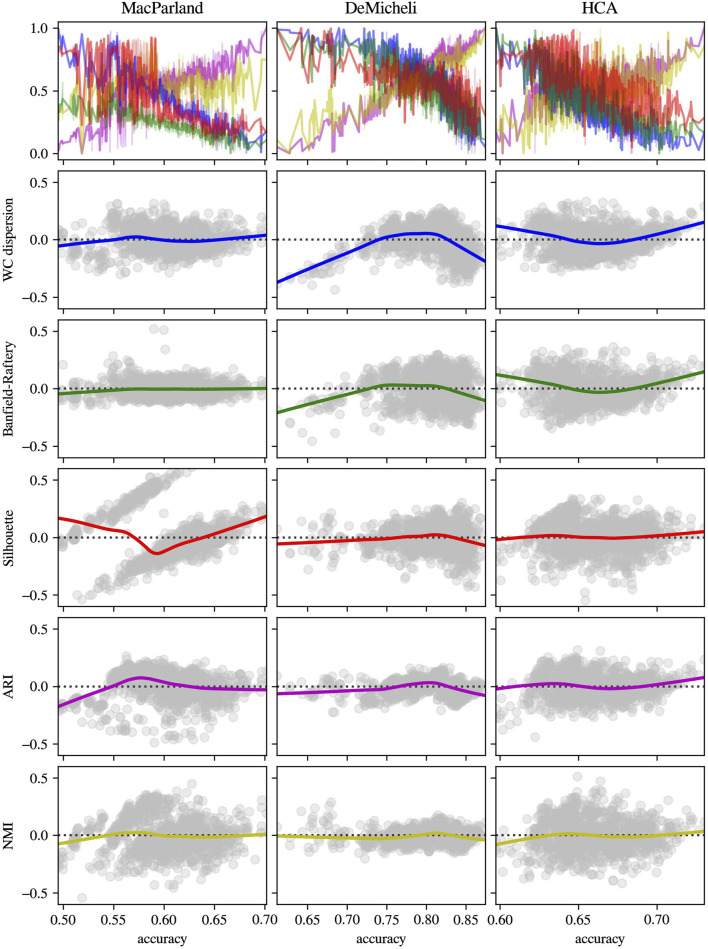
Distribution and residual plot of intrinsic and extrinsic metrics for DESC algorithm outcomes. For each dataset (in columns), intrinsic measures (Banfield-Raftery in green, WC_dispersion in blue and Silhouette index in red) and extrinsic metrics (ARI in magenta and NMI in yellow) have been represented in terms of normalized distribution in function of accuracy (first line) and the scatterplot of the residual of linear regression.

To validate the consistency of those intrinsic metrics, the distribution of the Banfield-Raftery and WC_dispersion as a function of accuracy, alongside the respective deviation from linearity of each intrinsic measure, has been compared with two extrinsic measures (i.e., ARI and NMI), and with the Silhouette index, as the most commonly used intrinsic metric. The results in Figure 8 obtained from Leiden outcomes showed that, while the linear tendency of Banfield-Raftery and WC_dispersion is evident, the Silhouette index demonstrates a higher degree of divergence from linearity. Moreover, those intrinsic metrics are anticorrelated with extrinsic metrics. As demonstrated in Figure 9, the intrinsic metrics for DESC outcomes exhibited a heightened propensity of divergence from linearity, with varying extents observed across the diverse datasets.

## 4 Discussion

The primary objective of this study was to evaluate the impact of diverse clustering parameters, including the methodology employed for computing the neighborhood graph, distance metric, resolution, number of nearest neighbors, number of principal components, on the accuracy of the clustering process. The Leiden algorithm has been selected as the clustering method for determining the concordance of cells with their predefined cell types (i.e., the accuracy), both obtained with CellTypist to avoid any possible bias. Techniques based on community detection, like Louvain and Leiden algorithms, have been shown to outperform alternative data clustering algorithms across a range of evaluation criteria (Yu et al., 2022). Whereas the Louvain algorithm is no longer maintained, the Leiden algorithm is the core strategy of widely used methods for scRNA data clustering like Seurat, that has been recently recommended as the first choice for scRNA-seq clustering (Traag et al., 2019; Zhang et al., 2023). In addition to conventional clustering algorithms, the advent of deep learning has given rise to a variety of approaches based on neural networks. In the field of single-cell analysis, the Deep Embedding for Single-cell Clustering (DESC) method has exhibited superior performance metrics when compared to alternative approaches (Liang et al., 2024). Consequently, it has been selected as a representative of deep learning methods and allowed to evaluate further the impact of number of nearest neighbors and resolution.

### 4.1 Leiden vs. DESC

It was evident that both algorithms demonstrated a marked increase in accuracy when applied to the DeMicheli dataset, in comparison to their performance on other datasets. Nevertheless, the DESC algorithm exhibits inferior performance in comparison to the Leiden algorithm when applied to the MacParland and HCA datasets, despite the observation that both distributions demonstrate reduced variance. Whilst the diminished performance could be ascribed to the imperative to augment the investigated values for resolution in order to achieve enhanced sampling of clustering accuracy (as substantiated by the UMAP plot), the enhanced performance could be attributable to the fact that DESC balances the biological and technical disparities between clusters and mitigates the influence of batch effect (Liang et al., 2024).

Unlike Leiden, the DESC algorithm appears particularly sensitive to changes in parameter values: suboptimal configurations such as these give rise to a distinct cluster of low accuracy values, producing a bimodal distribution. As a result, standard parametric assumptions are partially violated, and even the robust regression model, although effective, tends to treat the left mode as a set of outliers, down-weighting its influence. This emphasizes how, in the case of DESC, poor parameter selection can severely deteriorate clustering performance, making the method more fragile than Leiden under misspecification. HCA shows a more regular distribution of residuals, though mild deviations persist.

Given the distribution of accuracy and the resampling approach employed for data generation, a robust linear mixed-effects regression model was utilized to model the data. Indeed, the robust approach is recommended in the presence of outliers (Santos, 2020).

It is noteworthy that, due to the stochastic nature of nearest-neighbor search algorithms, the introduction of different random seeds can slightly influence the final output. This is because the seed affects the initial steps of the algorithm that have random elements, such as the choice of initial points for modularity optimization or the order of exploration of nodes in the graph. These variations can lead to small divergences in the identified clusters, especially in complex datasets with many border zones between clusters or in the presence of cells that could be assigned to more than one cluster depending on the initial configuration of the algorithm. Through the analysis of the main effect model, it was determined that the variability introduced by varying the starting point of the cluster algorithm can be regarded as a secondary effect in comparison to the other variables therefore this stochastic effect could be ignored and is not considered further in this analysis.

### 4.2 Method and metric

An examination of the first-order interaction model revealed that the UMAP method exhibited a notable positive impact on clustering accuracy, as compared to the Gauss method, for two out of three datasets. Nevertheless, the positive effect is consistently significant when the Euclidean metric is employed in conjunction with the method, as evidenced by the results obtained on all three datasets. Therefore, even if the marginal effect would suggest that the cosine metric is generally preferable, the negative baseline impact of the Euclidean metric is largely offset by its positive interactions with UMAP method and higher resolution, making it a preferable choice for two out of three datasets. However, as demonstrated in Table 3, the cosine metric is to be preferred over the Euclidean metric with the UMAP method when all cells are utilised in order to achieve the highest possible accuracy. Therefore, the cosine metric could be advantage in case of higher number of cells.

### 4.3 Number of neighbors

The number of nearest neighbors modulates the structure of the nearest neighbors graph. An insufficient value may result in a fragmented view of the data, whereby the overall structure of the cells is lost, leading to the formation of numerous small clusters that may represent minor or random variations rather than biologically significant differences. Consequently, there is a risk of isolating cells or small groups of cells that, in reality, belong to larger cell populations and, therefore, are characterized by a greater number of connections in the graph. Conversely, an excessively high number of neighbors has the potential to link together cells that should remain distinct, thereby obscuring the distinction between different states or cell types (Dann et al., 2022). This can be particularly problematic in the presence of cell heterogeneity or transition states, where it is crucial to maintain the ability to distinguish between subtly different cell populations. The optimal number of nearest neighbors must therefore be selected in order to accurately capture the local and global structure of cells on which to implement clustering. This is reflected when a high number of nearest neighbors reduces accuracy when considered alone (for both Leiden and DESC algorithm) or in combination with the Euclidean metric. However, interactions with UMAP and high clustering resolution demonstrated that these negative effects can be mitigated, suggesting that the choice of the number of nearest neighbors should be considered in combination with other parameters.

In summary, lower values lead to sparser and more locally sensitive graphs, which better preserve fine-grained cellular relationships. This sparsity increases the effect of the resolution parameter, allowing both algorithms to detect small, tightly connected communities. In contrast, higher values of nearest neighbors promote more connected graphs, smoothing the manifold and reducing sensitivity to local structures—especially detrimental when combined with low resolution.

### 4.4 Resolution

The resolution thus determines the sensitivity of the clustering method in identifying cell subpopulations. At low resolution, the algorithm tends to produce a smaller number of clusters, each comprising a larger number of cells. This approach may be beneficial when the objective is to discern the predominant cell lineages present within a given sample, thereby reducing the likelihood of overly subdividing homogeneous populations. Conversely, an increase in resolution results in enhanced sensitivity, enabling the differentiation of more precisely delineated cell populations. This is particularly advantageous when searching for subpopulations or rare transition states, which may prove crucial for understanding specific biological processes or characterizing a particular disease. However, as was the case with the selection of the number of nearest neighbors, an excessively high value may result in the over-segmentation of the data, leading to the recognition of potentially biologically irrelevant clusters. In contrast, a value that is too low may fail to reveal the intrinsic complexity of the sample, with cells that are similar but belong to distinct biological states being aggregated together (Kiselev et al., 2019).

In summary, increasing the clustering resolution directly increases the number of clusters detected by both clustering algorithms, effectively “over-partitioning” the neighborhood graph. While this can lead to overfragmentation in datasets with few true clusters, it significantly improves cluster separation when the underlying cell populations are heterogeneous and abundant. This explains why the best configurations consistently include resolution = 2, regardless of dataset.

### 4.5 Number of principal components

The number of principal components plays a more nuanced role. Principal component analysis defines the latent space in which distances for the neighborhood graph are computed. The inclusion of an insufficient number of PCs may prove inadequate for accurately capturing the underlying biological complexity. This could result in a potential fusion or splitting of biologically distinct clusters. While increasing the number of components generally improves variance capture, it can also introduce noise and distort local neighborhood structures. Therefore, the optimal number of principal components to include represents a compromise between the necessity to reduce the complexity of the data while retaining sufficient information to accurately discriminate between different states or cell types (Stuart et al., 2019). When considered in isolation, its effect is therefore modest or non-monotonic. However, when paired with high clustering resolution and low number of neighbors, a higher number of principal components (e.g., 30 or 50) allows Leiden algorithm to exploit a richer representation of the data, enhancing the ability to resolve subtle substructures. This effect is dataset-specific—particularly beneficial in HCA and MacParland, and marginal in DeMicheli—highlighting that the utility of dimensional richness depends on the intrinsic structure of the data (e.g., number and dimension of clusters, distance between clusters).

As demonstrated, the impact of augmenting the number of principal components on accuracy is not consistent across the three datasets. This may be indicative of the fact that this parameter is significantly influenced by the inherent complexity of the data. In order to further explore this aspect and determine whether this complexity is perhaps tissue-specific, three additional datasets originating from the same anatomical districts were analysed using the robust linear mixed regression model. The extraction of the optimal and suboptimal parameter configurations for the three paired datasets (see Supplementary Table S7) demonstrated a complete overimposition of both parameter configurations for the liver datasets, and an almost complete overimposition for the kidney datasets (i.e., in this case the Muto dataset demonstrated a preference for the cosine metric over the Euclidian metric in comparison to the HCA). It is evident that there is a lack of congruence between the method, the number of principal components, and the nearest neighbours for datasets pertaining to skeletal muscle. Consequently, it is not possible to conclude that the best and worst configurations of parameters are tissue-specific. However, it is important to note that the number of clusters is likely to be a contributing factor in this phenomenon since both liver datasets exhibit a comparable number of clusters (i.e. 20 and 21 clusters, respectively), a similarity that is not observed in the other datasets.

The impact of the selected optimal and sub-optimal configurations for parameters, along with the significance of resolution, is particularly evident in the UMAP plots. In the representation of DESC outcomes misclassifications is clearly visible. For instance, Hepatic Stellate Cells are misclassified as periportal LSECs (MacParland), EC-AEA as EG-GC, NKT as NK (HCA), and CLDN5+PECAM1+Endothelial as ICAM1+SELE + VCM1+Endothelial (DeMicheli). In Leiden representation, the number of hepatocyte types from the MacParland dataset has been reduced by half. Furthermore, the distinction between inflammatory and non-inflammatory macrophages, as well as endothelial cells, has been eliminated. It is evident that, in the DeMicheli dataset, the endothelial cells are predominantly misclassified. This phenomenon may be explained by the heterogeneity of endothelial cells, which complicates the targeting of the endothelium. This challenge is further compounded by the paucity of knowledge regarding the identification and functional inventory of EC phenotypes (Pasut et al., 2021). The results of the HCA revealed that, in spite of the optimal configuration, the number of clusters detected was inadequate to accurately depict the complexity of the tissue.

### 4.6 Intra and cross-dataset assessment of intrinsic measures-based ElasticNet model

As a consequence of addressing the primary objective, it is now feasible to employ a range of accuracy values obtained through the implementation of diverse parameter configurations. In order to define a proxy for predicting the accuracy of cell clustering, fifteen intrinsic goodness metrics have been calculated for each of the aforementioned configurations. These metrics can be classified into two main categories: those based on intra- and inter-cluster pairwise distances and those based on dispersion measures. The correlation and hierarchical cluster analysis of the variables revealed the presence of consistent patterns, particularly a positive correlation between: Ball-Hall, and PBM; Banfield-Raftery, and WC_Dispersion; C-index and Mclain-Rao; Point-Biserial, Ray-Turi, and SD_Separation; Silhouette index and the Wemmert-Gancarski. A strong negative correlation has been found between Dunn and Xie-Beni index; and no correlation between TSS and other variables.

In light of these correlations, ElasticNet has been selected as a linear regression model with both l1 and l2 regularization of the coefficients. This combination permits the construction of a sparse model in which a limited number of weights are non-zero, as is the case with Lasso, while simultaneously preserving the regularization properties of Ridge. Training the model on the outcome obtained through Leiden algorithm for MacParland and HCA dataset yielded high performance, with an R2 value exceeding 0.8 and an RMSE value of 0.0002 on average. In contrast, the DeMicheli dataset demonstrated comparatively lower performance, with an R2 value of 0.64 and an RMSE value of 0.0005 on average. This discrepancy may be attributed to the disparate distribution of accuracy data, which is concentrated in a narrower interval in the latter case. The analysis of outcomes from DESC algorithms resulted in inferior model performances in terms of R^2^, but not for RMSE, for MacParland and HCA datasets, with R^2^ decreasing to 0.76 and 0.64, respectively. In contrast, the model that was trained on the DeMicheli outcome from the DESC model demonstrated an increase in R^2^ to 0.73.

Given the potential for overestimation of performance due to the intra-dataset approach, an alternative method was employed whereby one dataset was removed from the training set and used for testing in a cross-dataset approach. In this case, the performance of ElasticNet model applied to Leiden outcome decreases to R^2^ = 0.72, 0.54, and 0.71 for MacParland, DeMicheli, and HCA respectively. The low performance of the model in case of DeMicheli could be easily explained by the fact that the accuracy of the other two datasets used for training span in different range, that indeed provide higher performance for both of them when used as training set. Analogously, the performance of ElasticNet model applied to DESC outcome decreases to R^2^ = 0.61, 0.53, and 0.57 for MacParland, DeMicheli, and HCA respectively. Also the RMSE increases to values higher that 0.13.

It is therefore evident that the reliability of the ElasticNet model trained with all computed intrinsic measures, is contingent on both the employed algorithm and the dataset utilised during the training process. The trained model demonstrates a limited capacity for generalisation, as evidenced by the cross-dataset results, particularly with regard to the DESC outcomes. In consideration of the aforementioned factors, the ElasticNet results have been utilised to establish a ranking of the intrinsic metrics, thereby identifying those metrics that can provide a more accurate assessment of the accuracy, irrespective of the employed methods and dataset.

ElasticNet estimates the weights of features and performs feature selections, weighting to zero irrelevant features (Zou and Hastie, 2005). However, it is important to note that the weights dedicated to linear fitting are irrelevant to the correlation between features and their corresponding labels. Consequently, they are not appropriate for feature importance ranking. An approach that is usually employed to rank chosen features is described in (Yu et al., 2020). Subsequently, the features under consideration could be evaluated according to the frequency with which they are identified as relevant. In this study, the objective is to furnish a proxy of accuracy; consequently, it is essential to generalise the findings of built models. Therefore, features which have been consistently selected, irrespective of the training dataset or the clustering algorithm, have been prioritized since they are more effective in generalisation. In this perspective, of the 15 features, only two exhibited coherent ElasticNet weights across the three datasets, two algorithms and both in intra and cross dataset assessment. The variables identified as being of particular significance were Banfield-Raftery and WC_dispersion. The low negative coefficients estimated by ElasticNet suggest that the lower the values, the higher the accuracy predicted. This trend can be attributed to the fact that the WC_dispersion is defined as the variability or spread of data points within a cluster (Brusco et al., 2020). A low value suggests that the data points within each cluster are similar and cohesively grouped, while a high dispersion suggests that the data points are more scattered and less cohesive. On the other hand, the Banfield-Raftery index is a weighted sum of the logarithms of the traces of the within-group scatter matrix of each cluster. As it is based on the cluster variances, it demonstrates greater suitability when the clusters are hyperspherical but of varying sizes (Todeschini et al., 2024). Therefore its suitability could be due to greater sensitivity to internal variance within clusters, making it a more robust and appropriate measure in contexts characterized by the presence of clusters with varying sizes, as is the case in single-cell RNA-seq data.

It is important to note that these results cannot be immediately compared with other methods in literature, since intrinsic metrics have been previously used as an instrument to estimate the optimal number of clusters, even if the number of clusters do not fully reflect the quality of the selected label set (Liu et al., 2021). Taking into account this limitation, since the majority of literature employs the silhouette index (Cheng et al., 2019), a comparison was made between the aforementioned significant metrics and the silhouette index. Moreover, extrinsic measures as ARI and NMI have been computed in the same conditions to check the consistency of chosen intrinsic metrics. The results for Leiden outcomes demonstrated that, whilst the Banfield-Raftery, and WC_Dispersion variables exhibited a slight divergence from linearity, the silhouette index did not demonstrate a linear behaviour in comparison to the computed accuracy. This finding indicates that the silhouette index is less effective in its role as an intrinsic metric for an immediate comparison of different clustering parameter configurations. The findings pertaining to DESC outcomes indicated that all intrinsic metrics manifested elevated levels of divergence from linearity, particularly at lower values of accuracy. Nevertheless, in each case, the chosen intrinsic metrics appeared to be negatively correlated with the extrinsic metrics. This finding demonstrated that they could be used as proxies for accuracy, with the advantage of not necessarily knowing the actual cluster labels.

This study has potential limitations. It made use of manually annotated datasets, which meant that the quality control procedures, that are the standard preliminary steps of single-cell analysis, were not undertaken. This was because only the labels for high-quality cells were available. In future work, it would be beneficial to consider a cluster of cells deemed to be of insufficient quality in order to analyze additional parameters, such as the impact of the minimum acceptable number of counts per cell on clustering stability. This approach will also assist researchers in evaluating the efficacy of the parameters employed for cell elimination based on the minimum acceptable number of reads. Furthermore, the clustering methods have been evaluated using the Scanpy toolkit, and a different number of functions and methods would be available in the R environment. It would be beneficial for future research to extend the analysis to a larger and more diverse set of single-cell datasets. The incorporation of datasets representing a broader range of biological conditions could serve to further validate the robustness of the clustering methods and parameters.

## 5 Conclusion

This study permitted an investigation into the influence of diverse clustering parameters on the accuracy of single-cell RNA-seq data analysis. To this end, a robust linear mixed-effects regression model was fitted in order to correctly identify the key factors affecting clustering performance. The number of nearest neighbors, resolution, number of principal components, distance metric and neighbor definition method were found to have a significant impact on accuracy. The analysis of the first-order interaction model demonstrated that clustering accuracy can be interpreted as the outcome of a trade-off between clustering resolution capacity and signal-to-noise ratio. The resolution capacity—driven by the clustering resolution, and modulated by the number of nearest neighbors and principal components—determines the algorithm’s ability to separate distinct cellular populations. At the same time, increasing the number of principal components carries the risk of incorporating noisy or biologically irrelevant variation. Optimal configurations strike a balance between these forces, favoring high clustering resolution, low number of nearest neighbors, and a sufficiently expressive PCA space, especially when combined with UMAP, which preserve local geometries.

Among the intrinsic metrics used to predict accuracy, the WC_dispersion and Banfield-Raftery have been identified as those which demonstrate the most linear trend, negatively correlated with accuracy, irrespective of the dataset to which they are applied and the method used for clustering. Consequently, they can be utilised to evaluate the accuracy, with the benefit of not necessarily being aware of the actual cluster labels.

## Data Availability

Publicly available datasets were analyzed in this study. This data can be found here: The liver and skeletal muscles datasets have been downloaded from Gene Expression Omnibus (GEO) repository with the following accession numbers: GSE115469 and GSE143704. The kidney cortex data have been downloaded from https://explore.data.humancellatlas.org/projects/29ed827b-c539-4f4c-bb6b-ce8f9173dfb7.
