# Supplementary material for: Optimization of clustering parameters for single-cell RNA analysis using intrinsic goodness metrics
Source: Front Bioinform. 2025 Jun 11;5:1562410. doi: 10.3389/fbinf.2025.1562410 (PMC12187673; doi:10.3389/fbinf.2025.1562410)
Supplement: Supplementary file 1 [file DataSheet1.pdf]

Supplementary Material

1 ROBUST LINEAR MIXED REGRESSION MODEL

Robust linear mixed regression was implemented using the function `rlmer` from the R package `robustlmm` (Koller et al., 2016). The model aims to estimate the fixed and random effects associated with clustering accuracy across different configurations of key parameters used in neighborhood graph construction and clustering.

The fixed effects include five categorical variables:

- **Method** (m): Method for computing the neighborhood graph, with levels `Gauss` and `UMAP`. These methods influence how global and local cell structures are captured. In Scanpy, the nearest neighbor distance matrix and neighborhood graph are derived via UMAP or the Gauss kernel.
- **Metric** (met): Distance metric used to calculate pairwise distances in the reduced gene expression space. Options include `Cosine` and `Euclidean`. These metrics influence how similarity is computed when identifying nearest neighbors.
- **Number of principal components** (npc): Values include 10, 20, 30, and 50. Principal component analysis is used to project cells into a reduced-dimensional space while preserving as much variance as possible. This variable captures the impact of dimensionality reduction on clustering performance.
- **Number of nearest neighbors** (nn): Values include 10, 20, and 30. This parameter defines the size of the local neighborhood around each cell in the PCA-reduced space, which is used to construct the graph structure for downstream clustering.
- **Resolution** (res): Values include 0.5, 0.8, 1, and 2. This parameter governs the granularity of the clustering solution by controlling how fine or coarse the identified communities are.

Let $\boldsymbol{M}$ be the matrix of main effects (excluding the intercept):

$$\boldsymbol{M} = \begin{bmatrix} m_1 & res_1 & npc_1 & nn_1 & met_1 \\ m_2 & res_2 & npc_2 & nn_2 & met_2 \\ \vdots & \vdots & \vdots & \vdots & \vdots \\ m_n & res_n & npc_n & nn_n & met_n \end{bmatrix}$$

Let $\boldsymbol{Z}$ be the matrix of all first-order interactions among the variables in $\boldsymbol{M}$:

$$\boldsymbol{Z} = \begin{bmatrix} m_1 \times res_1 & m_1 \times npc_1 & \dots & nn_1 \times met_1 \\ m_2 \times res_2 & m_2 \times npc_2 & \dots & nn_2 \times met_2 \\ \vdots & \vdots & \vdots & \vdots \\ m_n \times res_n & m_n \times npc_n & \dots & nn_n \times met_n \end{bmatrix}$$

The complete model matrix is then:

$$\boldsymbol{X} = [\mathbf{1} \quad \boldsymbol{M} \quad \boldsymbol{Z}]$$

Let $\boldsymbol{y}$ be the response vector of clustering accuracy values:

$$\boldsymbol{y} = \begin{bmatrix} y_1 \\ y_2 \\ \vdots \\ y_n \end{bmatrix}$$

The fixed effects are expressed as:

$$\boldsymbol{X\beta} = \mathbf{1}\beta_0 + \boldsymbol{M\beta_M} + \boldsymbol{Z\beta_Z}$$

The random effects are defined over the grouping variable `adata`, which identifies each of the 100 independent stratified subsamples obtained by randomly selecting 20% of each cell type. This accounts for the variability introduced by repeated subsampling:

$$\boldsymbol{u}_{adata} \sim \mathcal{N}(0, \sigma_u^2 \boldsymbol{I})$$

The full model is therefore:

$$\boldsymbol{y} = \boldsymbol{X\beta} + \boldsymbol{u}_{adata} + \boldsymbol{\epsilon}, \quad \text{with} \quad \boldsymbol{\epsilon} \sim \mathcal{N}(0, \sigma_\epsilon^2 \boldsymbol{I})$$

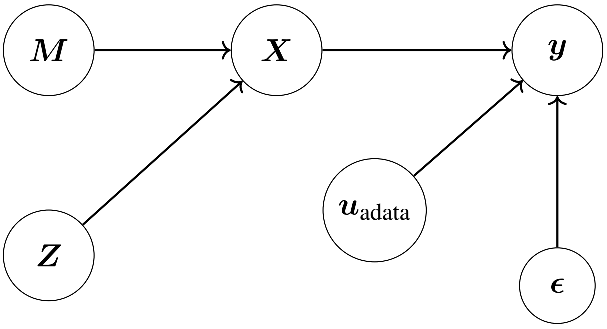

Figure S1: Graphical summary of the robust linear mixed model structure.

2 MODEL DIAGNOSTICS

The following figures show the diagnostics on the robust mixed linear regression model for the MacParland, DeMicheli and HCA datasets and for the Leiden and DESC algorithms, via Normal Q-Q and Residual mean.

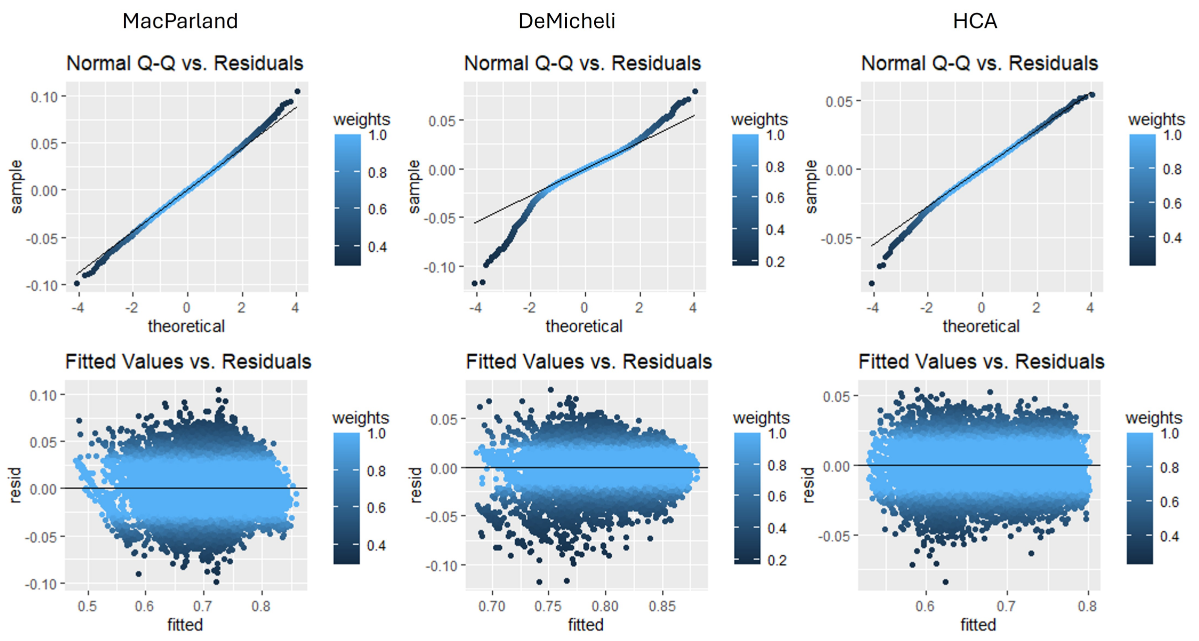

Figure S2: Q-Q plots and residual mean of the Robust Linear mixed regression models on MacParland, DeMicheli, and HCA accuracy using the Leiden algorithm

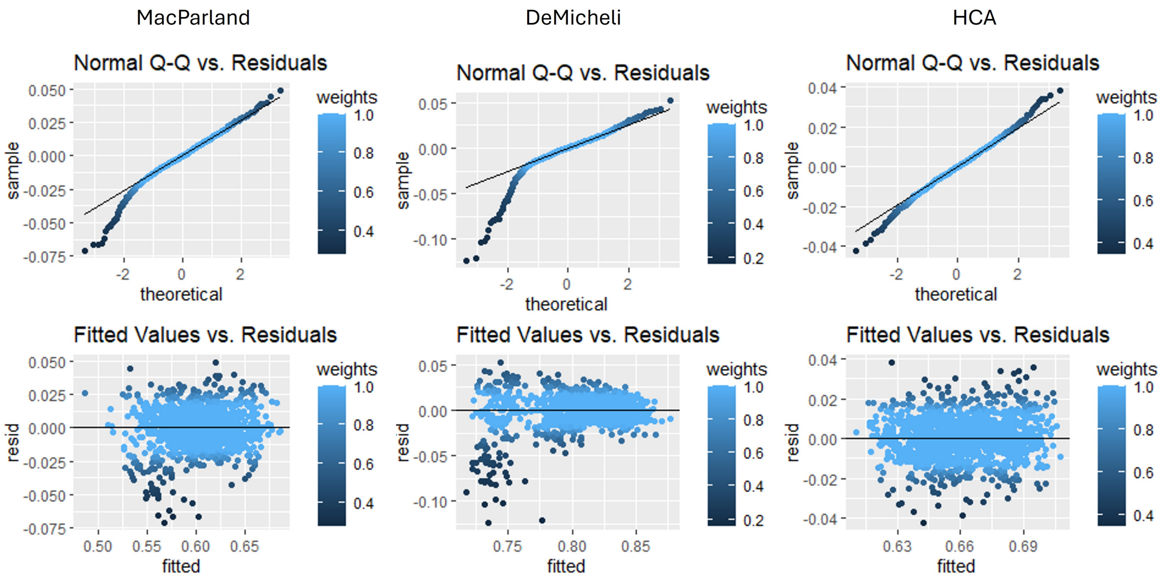

Figure S3: Q-Q plots and residual mean of the Robust Linear mixed regression models on MacParland, DeMicheli, and HCA accuracy using the DESC algorithm

3 PARAMETER TABLES

The following tables show the robust mixed linear regression parameter estimates for the MacParland, DeMicheli and HCA datasets and for the Leiden and DESC algorithms.

Table S1. Estimated regression parameters for the MacParland dataset under the Leiden clustering configuration

Parameter	Estimate	Std. Error	t value	Significant
(Intercept)	0.6526	0.0017	383.1	*
nn20	-0.0282	0.0012	-22.7	*
nn30	-0.0456	0.0012	-36.6	*
npc20	0.0512	0.0014	37.8	*
npc30	0.0311	0.0014	22.9	*
npc50	-0.0080	0.0014	-5.9	*
meteuclidean	-0.0123	0.0011	-11.4	*
res0.8	0.0170	0.0014	12.6	*
res1	0.0212	0.0014	15.7	*
res2	0.0328	0.0014	24.2	*
mumap	0.0011	0.0011	1.0	
nn20:npc20	-0.0124	0.0012	-10.5	*
nn30:npc20	-0.0235	0.0012	-20.0	*
nn20:npc30	-0.0203	0.0012	-17.3	*
nn30:npc30	-0.0370	0.0012	-31.5	*
nn20:npc50	-0.0292	0.0012	-24.8	*
nn30:npc50	-0.0504	0.0012	-42.9	*
nn20:meteuclidean	-0.0067	0.0008	-8.1	*
nn30:meteuclidean	-0.0104	0.0008	-12.6	*
nn20:res0.8	0.0056	0.0012	4.8	*
nn30:res0.8	0.0074	0.0012	6.3	*
nn20:res1	0.0088	0.0012	7.5	*
nn30:res1	0.0119	0.0012	10.1	*
nn20:res2	0.0267	0.0012	22.7	*
nn30:res2	0.0355	0.0012	30.2	*
nn20:mumap	0.0170	0.0008	20.5	*
nn30:mumap	0.0322	0.0008	38.8	*
npc20:meteuclidean	-0.0114	0.0010	-11.9	*
npc30:meteuclidean	-0.0178	0.0010	-18.6	*
npc50:meteuclidean	-0.0228	0.0010	-23.7	*
npc20:res0.8	0.0281	0.0014	20.7	*
npc30:res0.8	0.0354	0.0014	26.1	*
npc50:res0.8	0.0422	0.0014	31.1	*
npc20:res1	0.0385	0.0014	28.4	*
npc30:res1	0.0570	0.0014	42.0	*
npc50:res1	0.0679	0.0014	50.1	*
npc20:res2	0.0533	0.0014	39.3	*
npc30:res2	0.0896	0.0014	66.1	*
npc50:res2	0.1302	0.0014	96.0	*
npc20:mumap	0.0180	0.0010	18.7	*
npc30:mumap	0.0265	0.0010	27.7	*
npc50:mumap	0.0364	0.0010	37.9	*
meteuclidean:res0.8	0.0082	0.0010	8.5	*
meteuclidean:res1	0.0137	0.0010	14.3	*
meteuclidean:res2	0.0260	0.0010	27.2	*
meteuclidean:mumap	0.0085	0.0007	12.6	*
res0.8:mumap	-0.0056	0.0010	-5.8	*
res1:mumap	-0.0074	0.0010	-7.7	*
res2:mumap	-0.0221	0.0010	-23.0	*

Table S2. Estimated regression parameters for the DeMicheli dataset under the Leiden clustering configuration

Parameter	Estimate	Std. Error	t value	Significant
(Intercept)	0.7801	0.0010	805.9	*
nn20	-0.0256	0.0008	-32.3	*
nn30	-0.0484	0.0008	-61.1	*
npc20	0.0525	0.0009	60.9	*
npc30	0.0297	0.0009	34.4	*
npc50	0.0075	0.0009	8.7	*
meteuclidean	-0.0268	0.0007	-39.3	*
res0.8	0.0324	0.0009	37.5	*
res1	0.0388	0.0009	45.0	*
res2	0.0465	0.0009	53.9	*
mumap	0.0207	0.0007	30.3	*
nn20:npc20	0.0051	0.0007	6.8	*
nn30:npc20	0.0081	0.0007	10.8	*
nn20:npc30	0.0028	0.0007	3.8	*
nn30:npc30	0.0039	0.0007	5.2	*
nn20:npc50	0.0007	0.0007	1.0	
nn30:npc50	0.0002	0.0007	0.3	
nn20:meteuclidean	-0.0039	0.0005	-7.4	*
nn30:meteuclidean	-0.0068	0.0005	-12.9	*
nn20:res0.8	0.0117	0.0007	15.6	*
nn30:res0.8	0.0221	0.0007	29.6	*
nn20:res1	0.0155	0.0007	20.8	*
nn30:res1	0.0276	0.0007	37.0	*
nn20:res2	0.0192	0.0007	25.7	*
nn30:res2	0.0384	0.0007	51.4	*
nn20:mumap	0.0073	0.0005	13.9	*
nn30:mumap	0.0153	0.0005	29.0	*
npc20:meteuclidean	0.0090	0.0006	14.8	*
npc30:meteuclidean	0.0076	0.0006	12.4	*
npc50:meteuclidean	0.0060	0.0006	9.9	*
npc20:res0.8	-0.0174	0.0009	-20.2	*
npc30:res0.8	0.0018	0.0009	2.1	*
npc50:res0.8	0.0118	0.0009	13.7	*
npc20:res1	-0.0190	0.0009	-22.0	*
npc30:res1	0.0019	0.0009	2.2	*
npc50:res1	0.0142	0.0009	16.4	*
npc20:res2	-0.0216	0.0009	-25.0	*
npc30:res2	0.0038	0.0009	4.5	*
npc50:res2	0.0205	0.0009	23.8	*
npc20:mumap	-0.0046	0.0006	-7.5	*
npc30:mumap	-0.0007	0.0006	-1.1	
npc50:mumap	0.0015	0.0006	2.5	*
meteuclidean:res0.8	0.0104	0.0006	17.1	*
meteuclidean:res1	0.0147	0.0006	24.1	*
meteuclidean:res2	0.0228	0.0006	37.4	*
meteuclidean:mumap	0.0058	0.0004	13.6	*
res0.8:mumap	-0.0158	0.0006	-25.9	*
res1:mumap	-0.0192	0.0006	-31.5	*
res2:mumap	-0.0279	0.0006	-45.7	*

Table S3. Estimated regression parameters for the HCA dataset under the Leiden clustering configuration

Parameter	Estimate	Std. Error	t value	Significant
(Intercept)	0.5980	0.0009	665.9	∗
nn20	-0.0194	0.0008	-24.6	∗
nn30	-0.0307	0.0008	-39.1	∗
npc20	0.0975	0.0009	113.9	∗
npc30	0.0937	0.0009	109.4	∗
npc50	0.0952	0.0009	111.1	∗
meteuclidean	-0.0217	0.0007	-32.0	∗
res0.8	0.0408	0.0009	47.6	∗
res1	0.0512	0.0009	59.8	∗
res2	0.0739	0.0009	86.3	∗
mumap	0.0091	0.0007	13.5	∗
nn20:npc20	-0.0000	0.0007	0.0	
nn30:npc20	0.0004	0.0007	0.6	
nn20:npc30	-0.0019	0.0007	-2.5	∗
nn30:npc30	-0.0022	0.0007	-3.0	∗
nn20:npc50	-0.0074	0.0007	-10.0	∗
nn30:npc50	-0.0105	0.0007	-14.2	∗
nn20:meteuclidean	-0.0034	0.0005	-6.6	∗
nn30:meteuclidean	-0.0056	0.0005	-10.7	∗
nn20:res0.8	0.0081	0.0007	11.0	∗
nn30:res0.8	0.0085	0.0007	11.5	∗
nn20:res1	0.0108	0.0007	14.5	∗
nn30:res1	0.0142	0.0007	19.1	∗
nn20:res2	0.0120	0.0007	16.2	∗
nn30:res2	0.0178	0.0007	24.0	∗
nn20:mumap	0.0066	0.0005	12.6	∗
nn30:mumap	0.0126	0.0005	23.9	∗
npc20:meteuclidean	0.0035	0.0006	5.7	∗
npc30:meteuclidean	0.0051	0.0006	8.4	∗
npc50:meteuclidean	0.0046	0.0006	7.6	∗
npc20:res0.8	-0.0187	0.0009	-21.9	∗
npc30:res0.8	-0.0088	0.0009	-10.3	∗
npc50:res0.8	0.0013	0.0009	1.6	
npc20:res1	-0.0225	0.0009	-26.2	∗
npc30:res1	-0.0123	0.0009	-14.4	∗
npc50:res1	0.0109	0.0009	12.8	∗
npc20:res2	-0.0084	0.0009	-9.8	∗
npc30:res2	0.0064	0.0009	7.4	∗
npc50:res2	0.0182	0.0009	21.2	∗
npc20:mumap	0.0012	0.0006	1.9	
npc30:mumap	0.0034	0.0006	5.6	∗
npc50:mumap	0.0074	0.0006	12.1	∗
meteuclidean:res0.8	0.0035	0.0006	5.8	∗
meteuclidean:res1	0.0084	0.0006	13.9	∗
meteuclidean:res2	0.0124	0.0006	20.4	∗
meteuclidean:mumap	0.0063	0.0004	14.7	∗
res0.8:mumap	-0.0052	0.0006	-8.5	∗
res1:mumap	-0.0103	0.0006	-16.9	∗
res2:mumap	-0.0114	0.0006	-18.8	∗

Table S4. Estimated regression parameters for the MacParland dataset under the DESC clustering configuration

Parameter	Estimate	Std. Error	t value	Significant
(Intercept)	0.5934	0.0019	306.2	∗
nn20	-0.0214	0.0021	-10.2	∗
nn30	-0.0477	0.0021	-22.7	∗
res0.8	0.0225	0.0021	10.7	∗
res1	0.0299	0.0021	14.2	∗
res2	0.0626	0.0021	29.8	∗
nn20:res0.8	0.0076	0.0030	2.6	∗
nn30:res0.8	0.0062	0.0030	2.1	∗
nn20:res1	0.0091	0.0030	3.1	∗
nn30:res1	0.0143	0.0030	4.8	∗
nn20:res2	0.0041	0.0030	1.4	
nn30:res2	0.0104	0.0030	3.5	∗

Table S5. Estimated regression parameters for the DeMicheli dataset under the DESC clustering configuration

Parameter	Estimate	Std. Error	t value	Significant
(Intercept)	0.7808	0.0017	473.4	∗
nn20	-0.0313	0.0021	-15.2	∗
nn30	-0.0456	0.0021	-22.1	∗
res0.8	0.0327	0.0021	15.8	∗
res1	0.0454	0.0021	22.0	∗
res2	0.0681	0.0021	33.0	∗
nn20:res0.8	0.0179	0.0029	6.2	∗
nn30:res0.8	0.0238	0.0029	8.2	∗
nn20:res1	0.0149	0.0029	5.1	∗
nn30:res1	0.0242	0.0029	8.3	∗
nn20:res2	0.0248	0.0029	8.5	∗
nn30:res2	0.0343	0.0029	11.8	∗

Table S6. Estimated regression parameters for the HCA dataset under the DESC clustering configuration

Parameter	Estimate	Std. Error	t value	Significant
(Intercept)	0.6456	0.0014	471.1	∗
nn20	-0.0079	0.0016	-5.0	∗
nn30	-0.0170	0.0016	-10.8	∗
res0.8	0.0197	0.0016	12.5	∗
res1	0.0297	0.0016	18.9	∗
res2	0.0449	0.0016	28.5	∗
nn20:res0.8	-0.0050	0.0022	-2.3	∗
nn30:res0.8	0.0006	0.0022	0.3	
nn20:res1	-0.0050	0.0022	-2.2	∗
nn30:res1	-0.0025	0.0022	-1.1	
nn20:res2	0.0047	0.0022	2.1	∗
nn30:res2	0.0101	0.0022	4.6	∗

4 TISSUE COMPARISON

In order to further explore the data-complexity effect and determine whether this complexity is perhaps tissue-specific, three additional datasets originating from the same anatomical districts were analysed using the robust linear mixed regression model. In particular, the following datasets have been downloaded from the CellTypist database (Domínguez Conde et al., 2022), along with their ground truth annotations:

- **Aizarani et al. [GSE124395]**: performed the single-cell RNA sequencing of 10.352 cells from normal liver tissue from nine human donors to construct a human liver cell atlas. This dataset consists of 21 clusters.
- **He et al. [GSE159929]**: performed single-cell transcriptomes of 84.363 cells derived from 15 tissue organs of one adult donor. Among these cells, 5.732 are from Skeletal muscle and are charcaterized by 10 clusters.
- **Muto et al. [GSE151302]**: performed single nuclei RNA-seq on 5 human adult kidney cortex samples obtaining 19985 cells. This dataset consists of 21 clusters.

Table S7. Best and worst configurations for Leiden algorithm.

Dataset	**Type**	**met**	**m**	**npc**	**nn**	**res**	**Accuracy**
Aizarani (Liver)	Best	cosine	umap	50	10	2	0.645 ± 0.001
	Worst	euclidean	gauss	50	30	0.5	0.497 ± 0.001
He (Skeletal Muscle)	Best	euclidean	gauss	10	10	2	$0.796 \pm 3.3\mathrm{e}^{-4}$
	Worst	euclidean	gauss	50	30	0.5	$0.732 \pm 3.3\mathrm{e}^{-4}$
Muto (Kidney)	Best	cosine	umap	50	10	2	$0.934 \pm 3.4\mathrm{e}^{-4}$
	Worst	euclidean	gauss	10	30	0.5	$0.860 \pm 3.4\mathrm{e}^{-4}$
